# New Subperiosteal Dental Implant Design with Finite Element Analysis and Mechanical Validation: A Design Validation Study

**DOI:** 10.3390/ma18030622

**Published:** 2025-01-29

**Authors:** Vicente Vanaclocha, Carlos Atienza, Amparo Vanaclocha, Andrés Peñuelas, Juan Gómez-Herrero, Francisco Pérez-Carrió, José Antonio Diego-Leyda, Nieves Sáiz-Sapena, Leyre Vanaclocha

**Affiliations:** 1 Department of Surgery, Medical School, University of Valencia, 46010 Valencia, Spain; 2 Instituto de Biomecánica (IBV), Universitat Politècnica de Valencia, 46022 Valencia, Spain; carlos.atienza@ibv.upv.es (C.A.); andres.penuelas@ibv.org (A.P.); juan.gomez@ibv.org (J.G.-H.); 3 Fresmedical Custom Digital Health, 03750 Pedreguer, Spain; paco@fresdental.es (F.P.-C.); jose.diego@fresdental.es (J.A.D.-L.); 4 Hospital General Universitario de Valencia, 46014 Valencia, Spain; nssapena@hotmail.com; 5 Medius Klinik, Ostfildern-Ruit Klinik für Urologie, 73760 Ostfildern, Germany; leyrevanaclocha@hotmail.com

**Keywords:** subperiosteal dental implant, titanium alloy, severe bone resorption, custom-made implant, finite element analysis, laser-powder bed fusion

## Abstract

New subperiosteal dental implants were designed to offer new options to edentulous patients with severe bone resorption for whom endosseous dental implants are not advisable. In our study, we aimed to design and manufacture subperiosteal dental implants with a minimum volume to facilitate surgical maneuvers and metal coverage by mucosa while ensuring maximal long-term implant strength and functionality. With cone-beam CT-scan data obtained from an edentulous patient, a maxilla and mandible recreation were created, and subperiosteal implants were designed and analyzed with FEA (250 MPa infinite-life limit stress). We redesigned them until they stood the infinite-life limit loads mentioned above. Then, they were manufactured with Ti6Al4V alloy and laser-powder bed fusion technology. All implants withstood mechanical tests (450 N static and 150 N loads for five-million cycle 150 N fatigue tests) with no failures. The first design resulted in maxillary and mandibular implant failures. Through the redesign process, the implant volume was reduced, and the number and placement of bone fixation screws were optimized while maintaining resistance to chewing. Once manufactured, these new implants withstood the loads mentioned above without failure. Our subperiosteal dental implants are an option for edentulous patients with severe maxilla and mandibular bone resorption. Manufactured with Ti6Al4V alloy and laser-powder bed fusion technology, they withstood the above-mentioned mechanical tests without failure.

## 1. Introduction

Endosseous dental implants are currently available as a solution for the loss of teeth [1]. They can be applied to most patients, except to those with insufficient bone at the maxilla or mandible, be it due to prolonged edentulism [2,3], local cancer [4,5,6], or general conditions, like severe osteoporosis, diabetes mellitus, or rheumatoid arthritis [7,8]. In this subgroup of patients, endosseous dental implants cannot be used as there is not enough remaining bone to support them [9]. In such cases, there are a number of surgical techniques to improve the amount and quality of the bone at the maxilla ridge and the mandible. They imply a surgical procedure to implant the bone graft, coming most of the time from the autologous iliac crest [10] or calvaria [11], and a prolonged waiting time for the graft to generate enough local bone to support the endosseous dental implants. Unfortunately, this goal cannot always be achieved. Additionally, some patients present unwanted results, like infections and further bone resorption [12,13].

Instead, in this subgroup of patients, a subperiosteal dental implant is another treatment option to consider [8]. Introduced by Dahl [14] in 1943, it entailed a surgical procedure to expose the remaining maxillary or mandibular bone by elevating wide oral mucosa flaps and undertaking direct complex intraoral bone measurements. Then, the mucosa was sutured back in place. The next step was to create a mold and manufacture the implant by casting with Vitallium (a cobalt–chromium alloy) or Tantalum. Finally, in a second surgical procedure, the device was implanted, the mouth mucosal flaps were elevated again, and the subperiosteal dental implant was screwed to the underlying bone. Aside from the technical difficulties of creating custom-made implants for the maxilla or mandible, the Vitallium did not integrate into the bone [15]. Mid- and long-term problems were frequent, with inadequate fitting, implant exposure, and local infection that resulted in its removal [16]. Goldberg and Gershkoff, in 1945, created a new subperiosteal implant made out of cobalt–chromium–molybdenum, adding an external oblique ridge to Dahl’s previous design [17]. In 1950, Lee used fewer support points over the ridge crest using a direct impression method [18]. Bodine, in 1953, placed the screw holes in the areas with the greatest bone strength and thickness [19]. In 1975, Small et al. [20] introduced a technique devised for patients with extensive mandibular bone atrophy. It entailed a submental skin incision with a transosseous implant insertion that was called the mandibular staple bone plate. This technique proved to be rather aggressive and did not achieve widespread use.

The initial dental implants were made out of iridio-platinum [21], Vitallium (chromium–cobalt alloy) [18], stainless steel [18], cobalt–chromium–molybdenum [17], and a crystallized material composed mainly of aluminum [22]. These implants did not achieve osseointegration, so eventually, they became loose or were the source of local infection. The first to introduce the titanium dental implants was Brånemark in 1978 [23]. When conducting a research project on the femurs of rabbits, he noticed that, upon inserting a hollow cage made out of titanium, the bone grew inside it to such an extent that, in case of a fracture, the bone would fracture first at the bone–titanium interface. Ever since then, titanium has been the metal of choice for dental implants [24].

However, manufacturing dental implants by machining or forging is costly and is cannot be customized to every patient [25]. Today, the available technology allows dental implant manufacturing to be precise and non-invasive. Three-dimensional patient-customized implants are made out of titanium alloy (Ti6Al4V), with excellent bone osseointegration and highly successful implant survival results [8,26,27].

Different research groups have created subperiosteal dental implants, mainly for the maxilla [2,3,8,26,27,28] and some for the mandible [3,7,29]. Yet, most designs include two independent pieces for each side of the maxilla [2,3,7,27,30] or for the mandible [3,7,29]. These separate metal constructs provide dental implants to a limited area, not to the whole maxilla or lower jaw. So, there is room for improvement.

Some FEA (finite element analysis) studies have evaluated the differences between dual- and mono-subperiosteal dental implants (one implant for each side of the maxilla or mandible versus a single implant for both sides) [28]. They report that mono-subperiosteal implants provide the best immediate results. Still, long-term dual ones offer better local accommodation and the best long-term implant survival. Further FEA studies are needed to optimize implant design, shape, and the number and location of fixation screws.

While zygomatic implants and bone grafting remain viable options in patients with severe maxillary or mandibular bone atrophy, subperiosteal plates stand out for their adaptability, patient-centric design, and reduced surgical burden [5,31]. Their customizability through CAD/CAM technology expands their stability and long-term success [5,25]. Future studies should explore their outcomes in terms of patient satisfaction, functional rehabilitation, and cost-effectiveness. Wishing to offer new options, our research group designed new subperiosteal dental implants for the maxilla and the mandible. With FEA and mechanical tests, the implants were analyzed, improving their design to optimize their mechanical resistance. The group strived for a minimum implant volume to facilitate surgical maneuvers and mucosa metal coverage while ensuring maximal long-term implant strength and functionality. Finally, implant shape, width, thickness, bone fixation screw number, and position were optimized to improve the patient’s masticatory function and aesthetics without compromising implant resistance.

This article aims to present our experience in the design and manufacturing of custom-made dental implants, hoping that this could be of help in the treatment of edentulous patients.

## 2. Material and Methods

**Figure 1:**
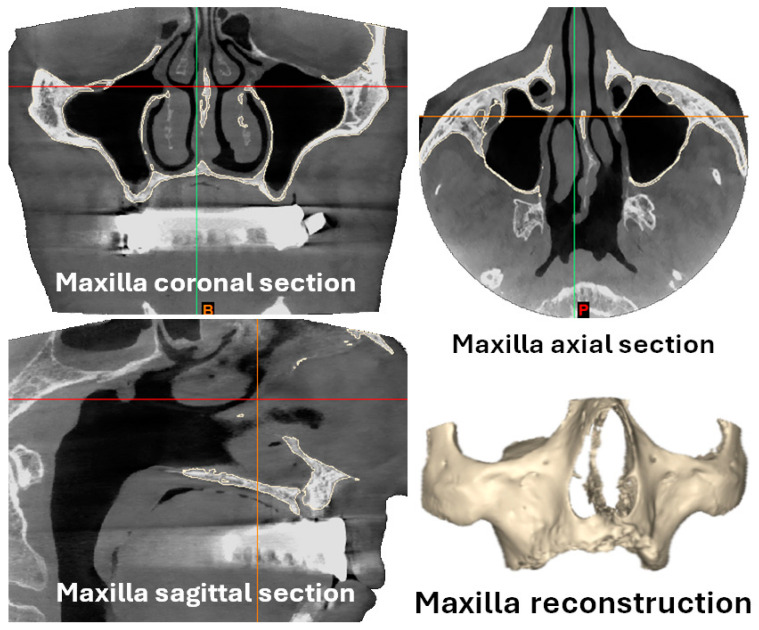

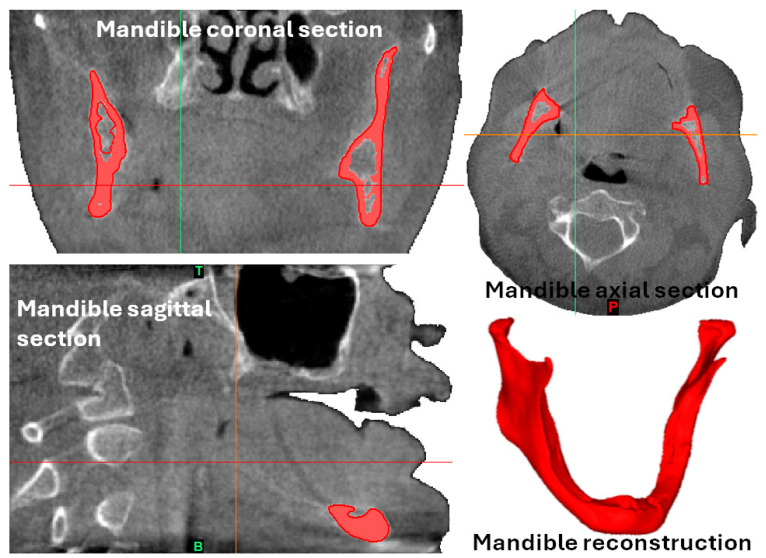
Patient CT-scan images with maxilla and mandible reconstructions.

The first step was obtaining the configurations of the maxilla and mandible from a 73-year-old male with severe maxillary and mandibular bone resorption (Cawood–Howell grade V) [32] with cone-beam CT-scan images. He had lost all his teeth due to tooth decay four years earlier. He was the average type of patient we see in our daily practice with this specific dental problem. The patient did not smoke, took no biphosphonates, and had no past medical history of cleft palate (Figure 1). The bone geometries (STL format or Stereolithography) were extracted from the CT-scan images by Fesmedical Custom Digital Health (FCDH).

Next, the FCDH processed the images into 3D objects using MIMICS software (Materialise, Leuven, Belgium, https://www.materialise.com/en/healthcare/mimics?gad_source=1&gclid=CjwKCAiA-ty8BhA_EiwAkyoa3_NiitbAPbvudArtKS3xs8HhYQYLAY4NwLBnsFrwIAYoQkmRbsX3AhoCl2oQAvD_BwE, (accessed on 28 December 2024)).

**Figure 2:**
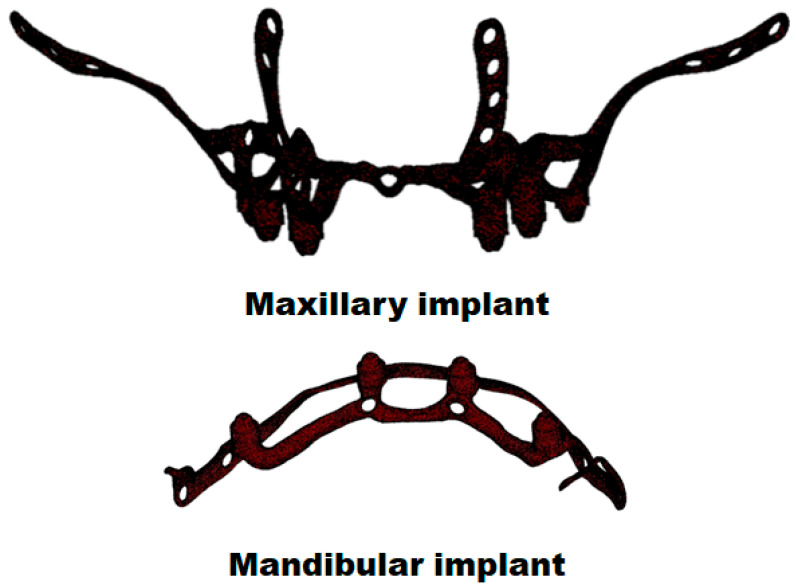
First implant designs for the maxilla and mandible with the 3-MATIC custom implant design program (Materialise, Leuven, Belgium).

**Figure 3:**
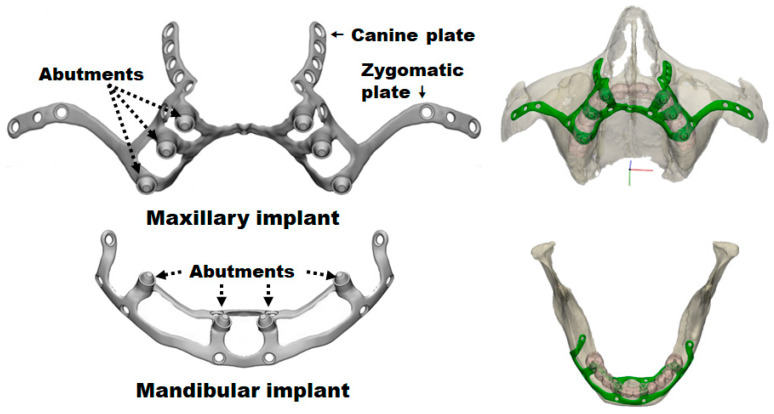
First implant designs for the maxilla and mandible.

The group took these geometries to the 3-MATIC custom implant design program (Materialise, Leuven, Belgium) and designed the implants (Figure 2). These were also exported as STL (Stereolithography) and meshed to tetrahedral elements. Finally, FCDH designed a subperiosteal dental implant plate for the maxilla and another for the mandible with Solid Works 2023 software (Waltham, MA 02451, USA) (Figure 3). This methodology segments the external cortex of the maxilla and the mandible, and based on this information, the 3D geometry of the plate was generated. The aim is to maintain maximum bone–plate contact to allow a better load transmission from the plate to the bone. In this way, overloading the plate is avoided at all times.

The Instituto de Biomecánica of Valencia (IBV) evaluated the implants with ANSYS 21^®^ software (Ansys, Inc., Canonsburg, PA 15317, USA), carrying out meshing and boundary conditons. The mesh was made using four-node tetrahedral elements with a total number of elements of 10,180. The boundary conditions were the embeddings at the connection points of the plate to the bone through the screws. The rest of the plate had no fixation pieces or elements in contact with the bone. The loads were applied in a distributed manner at the attachment of the plate to the dental bridge.

As the implants were custom-made, they were not entirely symmetrical for both sides of the mouth (Figure 3). The maxillary implant had two upgoing wings on each side: one that would rest on the canine and the other on the zygomatic bone buttresses. This shape should ensure that the fixation screws are inserted in the strongest remaining maxillary bone areas. Meanwhile, the mandibular implant covered the mandibular crest, both the front and back, with a sagittal section simulating a saddle.

The implants had abutments to apply chewing loads during the test and hold the dental prosthesis. These abutments had an equivalent geometry for a connection-type Nobel Multiunit RP (Nobel Biocare Services AG, Zürich-Flughafen, Switzerland) and an M1.4 thread for the fixation of the dental prosthesis with a dental screw. After surgery, these connecting pillars would emerge from the gum, allowing the fixation of the artificial denture.

The IBV created an FEA based on the data provided by Carnicero et al. [30] using tetrahedral elements and a parabolic shape for the meshing (Figure 3) with ten nodes and an average size of 0.25 mm (Table 1). To perform this, the IBV respected the original STL mesh (surface) and meshed it again with a maximum element size of 0.3 mm, an average of 0.25 mm, and at least three element layers with an average implant thickness of 0.8 mm.

Once the implants were meshed, the FEA was performed with ANSYS. The IBV performed two iterations for each implant type, which we will detail later.

**Figure 4:**
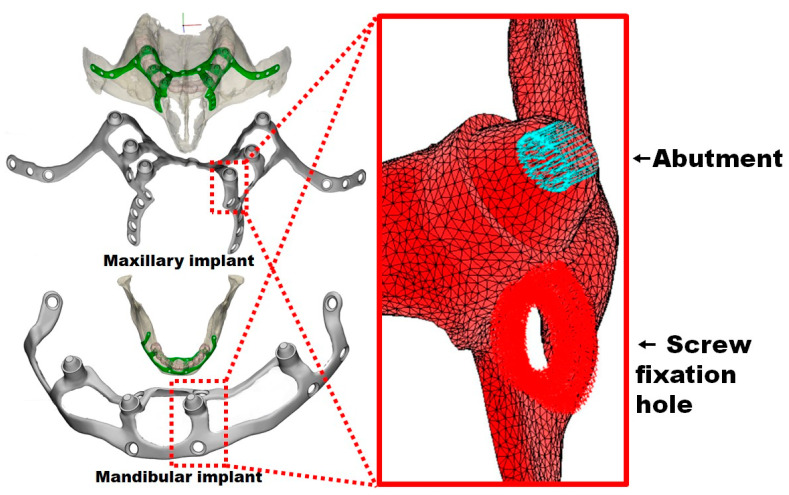
Implant FEA meshing with the fixation screw holes and the abutments.

The IBV applied a vertical load of 150 N chewing force and distributed it equally between the abutments on each implant side, as recommended [33,34,35,36]. Figure 4 depicts the holes for the insertion of the fixation screws (green color) and the abutments where the load was applied (blue color). The distribution of the implant stresses was calculated according to the von Mises criterion, while those of the nearby bone were studied with the Rankine stress criterion.

**Figure 5:**
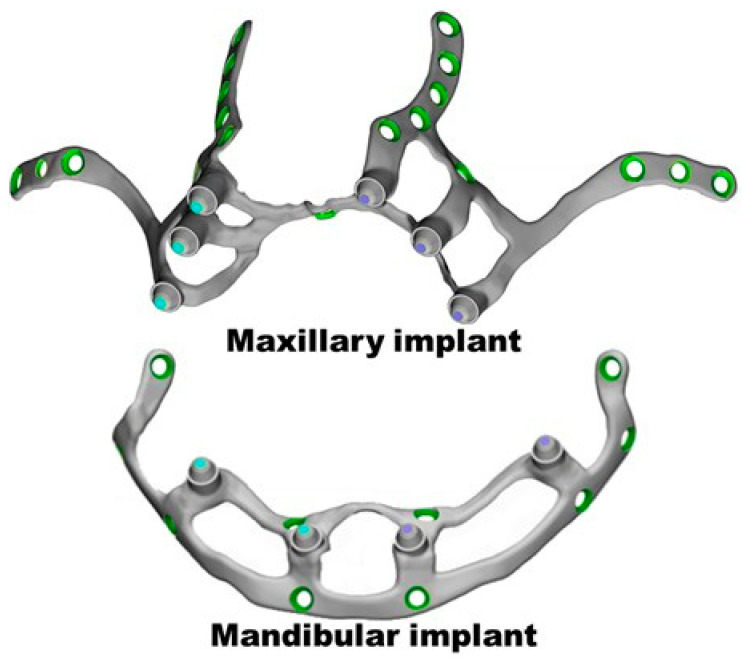
Implants with the points for fixation screw insertion (green color) and abutments to apply the loads (in blue and purple). The abutments are the places where the dental prosthesis will anchor.

**Figure 6:**
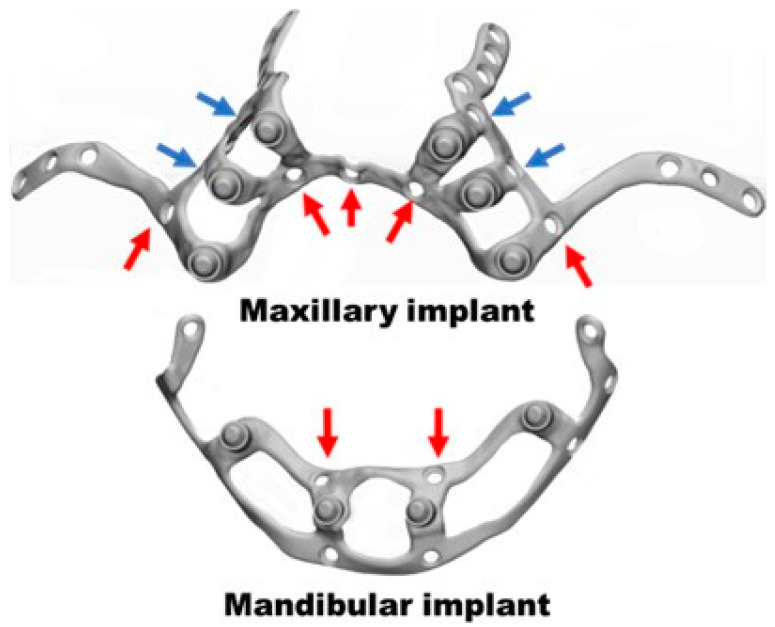
Second implant design changes for the maxilla and the mandible. The red arrows represent the new fixation screw holes, and the blue the ones with a new position.

With the FEA results of the first iteration, some areas needing improvement were identified. Accordingly, the implant designs were modified with 3-MATIC software (https://www.materialise.com/es/industrial/software/3-matic, (accessed on 28 December 2024)) (Figure 5). The implants’ shape and thickness were reduced or increased in some specific locations. The number of fixation points was increased (four for the maxillary and two for the mandibular implant) or changed in position (four for the maxillary implant) (Figure 6).

**Figure 7:**
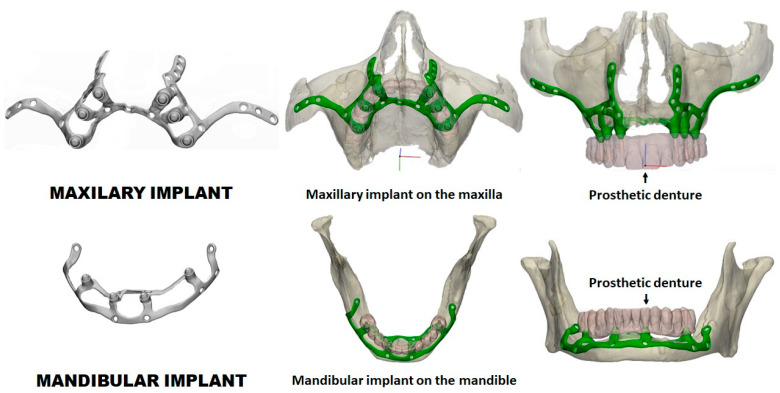
Final subperiosteal dental implant designs and how they will be applied with the dental prosthesis fixed to the abutments.

Then, the IBV repeated the FEA on the new designs to rule out any remaining problems. Figure 7 shows the final designs and how we applied them.

After confirmation that the new designs fulfilled our requirements, the next step was to manufacture the implants by FCDH. Fresdental, a specialized company in dental implant manufacturing, utilizes the Renishaw RenAM 500 3D printing system (RENISHAW, New Mills, Gloucestershire, UK) to produce high-quality titanium implants. The machine’s advanced capabilities ensure reproducibility, accuracy, and efficiency throughout the manufacturing process. RenAM 500 features a multi-laser system where all lasers can simultaneously focus on the entire powder bed. This flexibility reduces build times and optimizes laser energy utilization, making it an ideal solution for manufacturing precision implants. RenAM 500 employs a powder bed fusion (PBF) technique, where fine layers of titanium powder are fused using high-power lasers. Layer-by-layer fusion allows for the creation of highly intricate and precise geometries that match patient-specific designs. RenAM 500 undergoes regular calibration to maintain laser alignment and ensure uniform energy delivery. Laser technology was chosen for plate manufacturing over other methods, such as EBM, due to its ability to produce higher-quality parts with minimal need for subsequent polishing or machining work. After printing, the implants were removed from the build plate and subjected to additional treatments:

- Heat treatment: To relieve internal stresses and enhance mechanical properties.
- Surface finishing: To ensure smoothness and biocompatibility. This included polishing and sandblasting for optimal osseointegration.

Implants were inspected using metrology equipment to verify dimensions, tolerances, and surface quality.

### 2.1. Design of Maxilla and Mandible Recreations

The IBV obtained the maxilla and mandible configurations from the cone-beam CT-scan images of the patient mentioned above.

A gap was created between the maxillary and mandibular bone and the areas of the implant plates where the abutments lay, simulating significant bone resorption. Thus, the only contact between the bone and the subperiosteal implants was the spot where the fixation screws were located. Then, anchor plates were added to the maxilla and mandible recreations for fixation to an INSTRON E3000/863 (Norwood, MA, USA) universal testing machine.

**Figure 8:**
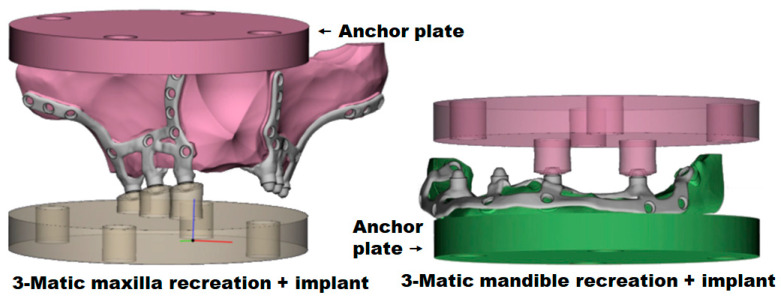
3-MATIC maxilla and mandible recreations and the subperiosteal dental implants with their abutments resting in place. A recess in the maxilla crest mimics total bone resorption, so the only support between the bone and implant would be the fixation screws.

Figure 8 shows the 3-MATIC maxilla and mandible recreations to test our subperiosteal dental implants with their abutments (places where the dental prosthesis would eventually be screwed).

With these data, the bone recreations were manufactured with RESIONE ANTI-IMPACT (Dongguan Godsaid Technology Co., Ltd., Guangdong, China) with 3-D LCD (Liquid Crystal Display) technology and an ANYCUBIC PHOTON MONO X 6K (Hongkong Anycubic Technology Co., Ltd., Hongkong, China) machine. This material is a nylon-like resin with extraordinary strength, tensile toughness, durability, fatigue tolerance, scratch-resistant surface, and high dimensional accuracy.

**Figure 9:**
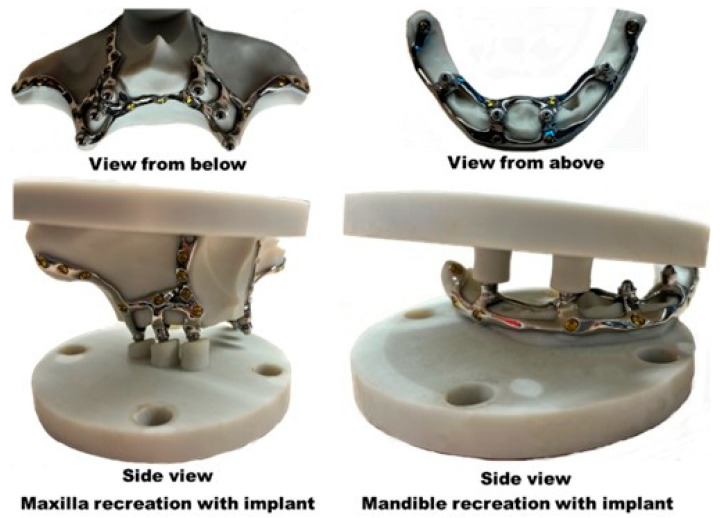
Maxilla and mandible nylon-like resin recreations with the subperiosteal dental implants in place.

Finally, the subperiosteal implants were mounted on the recreations of the upper and lower jaw using self-tapping screws (5 mm long and 2 mm in diameter) in a previously drilled hole 1.5 mm in diameter (Figure 9).

### 2.2. Mechanical Testing

Once the implant designs stood the intended loads in the FEA, FCDH manufactured them with L-PBF (laser-powder bed fusion) technology [37].

For the mechanical tests, four maxillary and four mandibular implants were manufactured with their corresponding maxilla and mandible recreations. For each type, one out of four underwent static loading, and the other three underwent fatigue testing. The IBV carried out all static and fatigue tests with the INSTRON E3000/863 (Norwood, MA 02062, USA) testing machine mentioned above, following the recommendations for titanium alloy dental implants manufactured by additive technologies [30]. Given the reported infinite-life limit of 250–300 MPa [38,39,40,41], we selected the lower range (250 MPa) for our study. We followed the recommendations indicated for materials manufactured by additive laser technologies, adding a safety factor on the maximum stresses that the implant should withstand. In the case of titanium, considering a breaking stress of 1000 MPa, we accepted that the stresses should not exceed 250 MPa at any point of the plate to ensure that it would not break due to fatigue. Under these conditions, it is estimated that the implant will never fail, provided these values are not surpassed [38].

**Figure 10:**
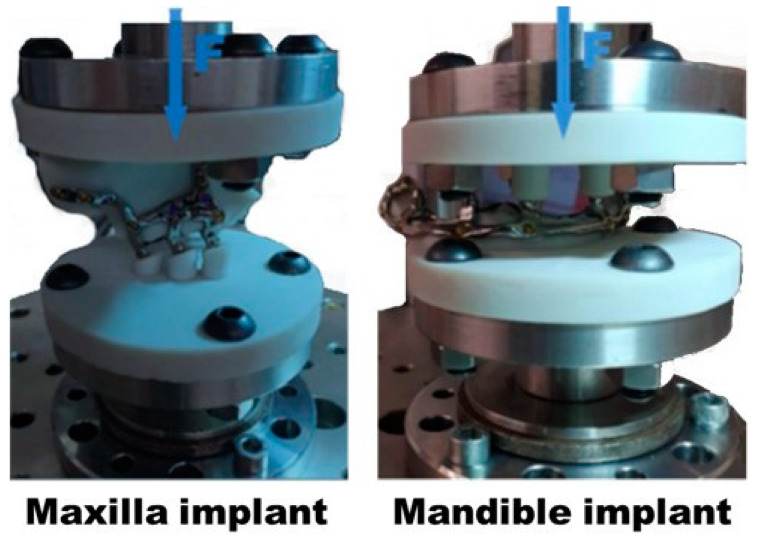
Maxilla and mandible implants and recreations in the INSTRON E3000/863 testing machine for testing.

For the tests, the maxilla or mandible recreations were attached to the testing machine actuator with their corresponding subperiosteal dental implants in place. The loads were applied by the actuator perpendicular to the plate by the INSTRON E3000/863 machine (Figure 10).

For the static tests, the end-of-test condition was sample failure or when 450 N was reached, whichever came first. The fatigue tests entailed cyclic compression sinusoidal 150 N loads (135 N amplitude and 15 N preload) with a 10 Hz load application frequency, with the end-of-trial condition being implant failure or reaching 5,000,000 cycles, whichever happened first. The test was carried out at 5 M cycles as indicated by the international standard for dental implants. This standard studies the useful life of dental implants through fatigue tests according to ISO 14801 [42] and UNE-EN ISO 14801 [43]. The standard estimates that 5 M cycles represent the chewing activity carried out in a minimum period of between 10 and 20 years of the patient’s life. The tests were carried out dry according to the previous standard.

### 2.3. Statistical Analysis

Free statistical analysis software R (R Development Core Team, Vienna, Austria, https://www.r-project.org/, (accessed on 28 December 2024)) was used in combination with the Deducer user interface [44].

## 3. Results

### 3.1. FEA Results

#### 3.1.1. First Iteration FEA Results

**Figure 11:**
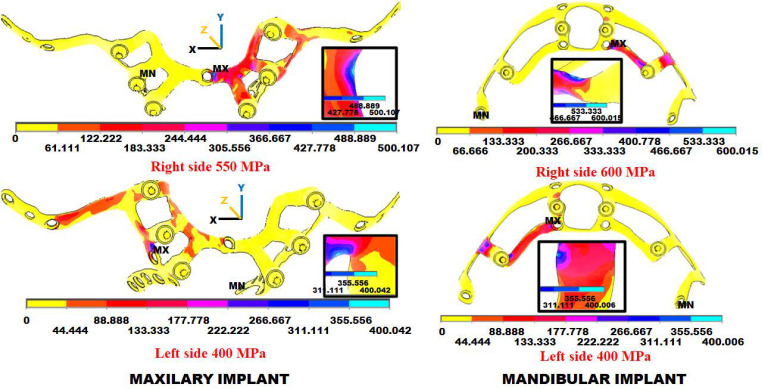
Stress (MPa) FEA results in the 1st iteration for the maxillary and mandibular subperiosteal dental implants with loads on the right- or left-side abutments with the maximum stress per chewing side. In red are the areas with the most considerable stress and, thus, with the highest risk of failure. The insets show the areas of maximum stress.

Figure 11 shows the stress per implant type and the chewing side. If the maximum stress value in any area of each implant is greater than 250 MPa, it runs the risk of notwithstanding fatigue tests, and it must be redesigned. This situation was the case with the first implant designs (red color areas), both for the maxilla and for the mandible, which were corrected by modifying the designs. The highest stresses were in the horizontal parts of the implants just above the abutments. In the maxillary implant, they reached 550 MPa for the right side and 400 MPa for the left. In the case of the mandibular implant, these stresses reached 600 MPa for the right side and 400 MPa for the left. In both cases, the stresses were well above the ones that could be accepted. All stress results shown from our FEAs are equivalent to von Mises stresses.

After analyzing the results of this first iteration, the recommendation was rounding all live edges, increasing the number of implant fixation screws, the implant width and thickness where it bears the highest stresses, and avoiding small radii between implant branches.

#### 3.1.2. Results of the Second Iteration FEA

**Figure 12:**
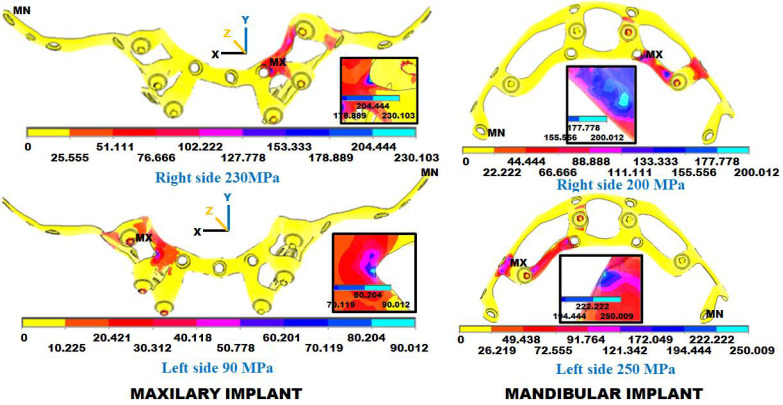
Stress (MPa) FEA results in the 2nd iteration for the maxillary and mandibular subperiosteal dental implants with loads on the right- or left-side abutments with the maximum stress per chewing side. In red are the areas with the most considerable stress and, thus, the highest risk of failure. Compared with the first iteration implants, the stress areas are reduced in size and can tolerate more considerable forces. The insets show the areas of maximum stress.

The FCDH made some design changes following the recommendations of the first iteration, and then the FEA was repeated, and the results were analyzed. Figure 12 shows the maximum stress for each implant type and chewing side, with a remarkable stress reduction due to implant design improvement. Now, no implant went over the 250 MPa, and again, the highest stresses were in the horizontal parts of the implants just above the abutments where the vertical chewing load was applied. In the maxillary implant, the stresses reached 230 MPa for the right side and 90 MPa for the left. Meanwhile, for the mandibular implant, they reached 200 MPa for the right side and 250 MPa for the left. In all cases, the stresses remained within the accepted limits.

The results of the second iteration recommend polishing the implants on all sides. These results involve removing excess material and rounding off all the sharp edges. Care was taken to minimize any reduction in implant width to maintain its structural integrity and functionality.

### 3.2. Implant Material Selection and Manufacturing

The IBV found out the mechanical and material properties of the L-PBF-manufactured Ti6Al4V alloy used to create our implants following the ASTM E8/E8M [45] and UNE EN ISO 6892-1:2020 [46] standards and the elastic modulus (118 GPa) and Poisson’s ratio (0.31). Both were similar to those reported by other research groups [28,30,47,48]. Table 2 shows the values for our samples, all above the reported ones. As the yield strength is 1007 MPa, a lower stress level would not cause any plastic or permanent deformation in the implant. FEA analyses corroborated that the stresses reached were less than the yield strength.

**Figure 13:**
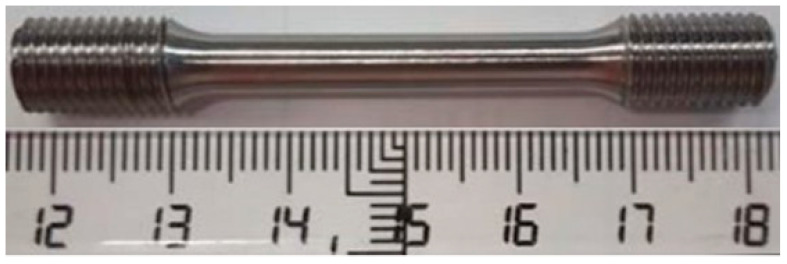
Sample for the mechanical tests of the Ti6Al4V alloy L-PBF used to manufacture our subperiosteal implants.

Five samples, like the one in Figure 13, were used in the static test. These samples had threaded heads for clamping to the testing machine and a diameter of 5 mm in the area to be tested in accordance with the UNE EN ISO 6892-1:2020 [49] standard.

The samples were created with a RenAM500 machine (RENISHAW, New Mills, Gloucestershire, United Kingdom) using 5 mm particle Ti6Al4V powder in continuous reuse and contributions of virgin powder. This technique is called the “infinite use strategy”, and the machine supplier recommends it for manufacturing environments. The use of virgin powder is deemed feasible only in the research but not in a production line due to the high costs of virgin titanium powder [50,51]. In any case, the oxygen content was below 0.13%, as requested for the Ti6Al4V ELI (grade 23) [1]. We measured it with the LECO ON 736 elemental analyzer (LECO, St. Joseph, Michigan 49085, USA) and the test method of fusion with inert gas according to ASTM 1409 [52]. Particle size distribution for the Ti6Al4V powder is shown in Table 3.

### 3.3. Mechanical Tests

The IBV first carried out the static tests and ensured that the implants supported the load at least three times, which would later be applied to the fatigue ones. All implants withstood the test, without any failures at a 450 N vertical load in the static tests and 150 N loads for 5 million cycles in the fatigue ones with no failures whatsoever.

## 4. Discussion

The experiment was designed to evaluate the efficacy of subperiosteal implants fabricated using advanced manufacturing techniques. The primary goals were:

1. Customization: To test the ability of additive manufacturing (AM), such as the RenAM 500 system, to produce implants tailored to patient-specific anatomy.
2. High Survival Rates: Subperiosteal implants have shown superior survival rates in cases of poor-quality bone compared to traditional endosseous implants and bone substitutes [53].

Our study proves that subperiosteal dental implants can be designed to the specific anatomical characteristics of the individual patient and later manufactured satisfactorily and made ready for clinical use.

Subperiosteal dental implants are currently considered for patients in whom endosseous dental implants are not an option due to insufficient maxillary or mandibular bone, especially if bone grafting techniques [54] are not advisable in this individual patient [7,8]. They have also been used successfully in the case of extensive bone resection due to oncological reasons [4,5].

The first subperiosteal implants did not even have osseointegration screws [24], but this is no longer the case. Titanium alloys are the preferred material for dental implants because they have excellent soft tissue tolerance [55] and osseointegration [55,56]. Once implanted in the craniofacial skeleton, they are commonly overgrown by nearby bone [57]. That is why they are widely used in dental implants [1], and we selected them for our implants.

The current 3D implant customization techniques have improved the long-term results [7,8,27,29,58]. However, local infections and mucosal atrophy with implant exposure are not uncommon [27,59]. Among the leading causes of failure are exposure to the titanium plate or poor placement during surgery, which results in the movement of the implant [60]. There are other options in the market, but many of our competitors are much thicker, with the surgical problems that this entails [5,55]. Our implants seek the minimum amount of metal with the thinnest possible thickness to minimize the risk of implant exposure due to a lack of adequate mucosa covering. We could reduce further the implant thickness, but the tensions supported would be higher and the risk of failure greater. Our methodology has found an optimal point between the use of the material and the risk of implant failure.

To improve the results further, some have added polymer guides to remove the residual bone contours that can lead to a suboptimal implant fitting but at the price of weakening the maxilla further [61]. Another option is to provide a greater implant roughness as it improves osseointegration but at the cost of worse fatigue behavior [38], particularly at the thinnest implant sections [62]. Fractures initiate at surface pores, voids, and partially unmelted titanium powder particles, causing failure over time [38,41]. As cracking begins on the metal surface, polishing is advised to achieve a surface roughness Ra ≤ 0.2 mm [38,41,63,64], as we did with our implants. Electron beam melting techniques elevate temperatures to 700 °C [37], enhancing metal quality and stress resistance [38]. The Hot Isostatic Pressing technique improves these properties further [38]. Meanwhile, L-PBF, which we used in our study, creates titanium implants that do not need any further finishing steps [48].

Another consideration is whether using virgin or recycled titanium powder makes any difference. The first type creates implants with a stronger fatigue resistance [41]. Conversely, recycling increases the oxygen content, improving roughness and osseointegration but decreasing fatigue resistance and elasticity [38,41,65,66]. For optimal results, the Ti6Al4V alloy oxygen content must remain under 0.13% [67], as was the case with our implants. The significantly increased cost of using virgin titanium powder makes this option unsuitable for clinical purposes [50,51]. An exception is the L-PBF manufacturing technique, the one we used in our study, as it provides excellent fatigue resistance with recycled Ti6Al4V powder [48,68].

The size of the titanium powder particles also matters, as 5 mm powder particles produce implants with better fatigue resistance than 2 mm ones [41]. That is why we used 5 mm-in-diameter titanium powder particles to manufacture our implants.

The relationship between implant design and performance is crucial. Duo ones, one for each side of the maxilla or mandible, support less stress on chewing [28]. Still, mono-implants have a maximum von Mises value of 129 MPa, well below the 250–300 MPa infinite-life limit [38,39,40,41]. Additionally, mono-implants behave better because they absorb and distribute local loads more evenly and allow the loading of the dental prosthesis much earlier [28]. Another advantage of a mono-implant is the greater ease of implantation in surgery [31]. As a final advantage, mono-implants are thinner than duo ones, facilitating successful covering by the oral mucosa, with less risk of implant exposure, and, thus, long-term failure due to repetitive infections [30]. Unfortunately, mono-implants require a more accurate fitting with precise positioning and alignment [28]. In our study, we used only mono-implants.

The screw number and position are essential to provide strong fixation to bone and increase long-term implant survival. If screws penetrate the Schneiderian membrane at the maxillary sinus, bacteria will colonize the metal implant over time, which will cause local infection with implant loosening and failure [8,26]. Most subperiosteal implants are anchored at the zygomatic and canine buttresses [7,8,28,30,61,69], where the bone is more robust, even in an atrophic edentulous maxilla. When such buttresses are not an option, perhaps due to a previous oncological resection, placement directly on the bony palate and anchoring it in the anterior nasal spine are options to consider [6].

There is some debate on how much load a subperiosteal implant should withstand to overcome long-term chewing forces in an atrophic edentulous maxilla or mandible. Some recommend that they should withstand 150 N vertical loads [30,33,34,35,36], with 50 N in the horizontal direction [28,30]. Others increase this vertical load tolerance to 200 N applied at a 45° angle because this angulation increases the von Mises stresses criterion to 270 MPa [26]. Others raise the vertical loads to 300 N [30,70,71] to overcome the implant infinite-life limit (250–300 MPa) [38,39,40,41]. In our study, vertical loads of 450 N were applied and no failures were seen. We reduced the implant size and thickness while maintaining the von Mises stresses criterion consistently below 250 MPa.

Our next step is a clinical study of the failure rate or problems in these implants compared to other types of dental implants.

## 5. Positive Aspects of the Proposed Medical Solution

The clinical use of subperiosteal plates allows for a greater number of plate fixations to the bone in those areas where the maxillofacial surgeon has determined that there is a greater bone density. Therefore, it is a new type of plate that, with good planning and selection of the areas, avoids placing the screws in areas of very low bone density and therefore ensures better fixation of the screws and greater long-term stability of the plate and the dental bridge.

On the other hand, this study has shown the importance of using FEA. It is a powerful technique that evaluates new implant designs in a non-invasive way. The initial designs were corrected according to the FEA results, and when satisfactory, the implants could be manufactured.

Finally, the plates were tested on the testing machine to validate the results obtained in the FEM models.

## 6. Limitations

As this design was tested on a single patient, the generalizability of the findings was limited. We plan to repeat the same study in more patients with similar maxilla and mandible bone resorption. The point left to study is the possible exposure of the implants and the infiltration of bacteria. Potential challenges are variations in patient anatomy or long-term implant exposure risks and bacterial infiltration. Clinical trials are pending.

## 7. Conclusions

With the final design, our maxillary and mandibular subperiosteal dental implants have met the FEA requirements. When manufactured, they withstood 450 N in the static and 150 N in the fatigue tests for five million cycles. These designs optimize the amount of titanium required to manufacture the implants, reaching an equilibrium between the decrease in the tensions supported and the increase in the use of the material without compromising their resistance to the chewing loads. In future studies, we will try to find this minimum amount of material, but this will entail a more significant number of design cycles and evaluations with FEA and mechanical tests.

## Figures and Tables

**Table 1:** The number of elements created with ANSYS 21^®^ software for each implant design model.

MODEL	ELEMENTS
1st type design 1	179.748
1st type design 2	125.321
2nd type design 1	209.490
2nd type design 2	140.755

**Table 2:** Tensile strength, elastic limit, elongation percentage, and elastic modulus on the different axes for the Ti6Al4V alloy used to manufacture our subperiosteal implants. MPa (Mega Pascals), GPa (Giga Pascals).

Sample	Tensile Strength (MPa)		Elastic Limit (MPa)		Elongation (%)		Elastic Modulus (GPa)	
	*X*-*Y* Axes	*Z* Axis	*X*-*Y* Axes	*Z* Axis	*X*-*Y* Axes	*Z*-Axis	*X*-*Y* Axes	*Z* Axis
T1	1085	1114	999	1049	12.8	15.3	120	119
T2	1078	1086	1010	1008	16.0	16.0	119	120
T3	1083	1080	1005	1027	11.6	16.4	116	123
T4	1081	1082	1013	1022	14.8	15.2	120	126
T5	1081	1088	1010	1025	12.4	18.4	114	119
MEAN	1082	1090	1007	1026	13.52	16.26	118	121
STD	2.60	13.78	5.50	14.75	1.81	1.29	2.68	3.04

**Table 3:** Particle size distribution for the Ti6Al4V alloy powder used. ASTM (American Association for Testing and Materials); μm (micron).

**Particle Size Distribution by Laser Diffraction (Microtrac) ASTM-8822**	**Volume%**	**Minimum** **μm**	**Maximum** **μm**	**Results** **μm**	**Meets Specifications**
	D10	FIO		26	N/A
	D50	FIO		37	N/A
	D90	FIO		54	N/A
	Size μm	Minimum%	Maximum%	Results%	Meets Specifications
	−20 μm	---	5	<2	YES
**Particle Size Distribution by Sieve Analysis ASTM-8214**	Size μm	Minimum Weight%	Maximum Weight	Results Weight%	Meets Specifications
	+75	---	0.0	0.0	YES
	+63	--	1.0	0.0	YES
	+53	---	5.0	0.4	YES

## Data Availability

The original contributions presented in the study are included in the article, further inquiries can be directed to the corresponding authors.

## References

[1] Nicholson J. (2020). Titanium alloys for dental implants: A review. Prosthesis.

[2] Arshad M., Khoramshahi N., Shirani G. (2023). Additively custom-made 3D-printed subperiosteal implants for the rehabilitation of the severely atrophic maxilla (a case report). Clin. Case Rep..

[3] Ângelo D.F., Vieira Ferreira J.R. (2020). The Role of Custom-made Subperiosteal Implants for Rehabilitation of Atrophic Jaws—A Case Report. Ann. Maxillofac. Surg..

[4] Garrido-Martínez P., Quispe-López N., Montesdeoca-García N., Esparza-Gómez G., Cebrián-Carretero J.-L. (2022). Maxillary reconstruction with subperiosteal implants in a cancer patient: A one-year follow-up. J. Clin. Exp. Dent..

[5] Cebrián Carretero J.L., Del Castillo Pardo de Vera J.L., Montesdeoca García N., Garrido Martínez P., Pampín Martínez M.M., Aragón Niño I., Navarro Cuéllar I., Navarro Cuéllar C. (2022). Virtual Surgical Planning and Customized Subperiosteal Titanium Maxillary Implant (CSTMI) for Three Dimensional Reconstruction and Dental Implants of Maxillary Defects after Oncological Resection: Case Series. J. Clin. Med..

[6] Watanabe T., Kawahara D., Inoue R., Kato T., Ishihara N., Kamiya H., Bessho K. (2022). Squamous cell carcinoma around a subperiosteal implant in the maxilla and the association of chronic mechanical irritation and peri-implantitis: A case report. Int. J. Implant. Dent..

[7] Nemtoi A., Covrig V., Nemtoi A., Stoica G., Vatavu R., Haba D., Zetu I. (2022). Custom-Made Direct Metal Laser Sintering Titanium Subperiosteal Implants in Oral and Maxillofacial Surgery for Severe Bone-Deficient Patients—A Pilot Study. Diagnostics.

[8] Van den Borre C., Rinaldi M., De Neef B., Loomans N.A.J., Nout E., Van Doorne L., Naert I., Politis C., Schouten H., Klomp G. (2021). Radiographic Evaluation of Bone Remodeling after Additively Manufactured Subperiosteal Jaw Implantation (AMSJI) in the Maxilla: A One-Year Follow-Up Study. J. Clin. Med..

[9] Di Stefano D.A., Arosio P., Capparè P., Barbon S., Gherlone E.F. (2021). Stability of Dental Implants and Thickness of Cortical Bone: Clinical Research and Future Perspectives. A Systematic Review. Materials.

[10] Wortmann D.E., Klein-Nulend J., van Ruijven L.J., Schortinghuis J., Vissink A., Raghoebar G.M. (2021). Incorporation of anterior iliac crest or calvarial bone grafts in reconstructed atrophied maxillae: A randomized clinical trial with histomorphometric and micro-CT analyses. Clin. Implant. Dent. Relat. Res..

[11] Etemadi Sh M., Tamizifar A., Aghajani F., Saei M. (2023). Reconstruction of Severely Atrophied Mandible and Simultaneous Implant Insertion with an Inverted Sandwich Technique. Case Rep. Dent..

[12] Beck F., Watzak G., Lettner S., Gahleitner A., Gruber R., Dvorak G., Ulm C. (2022). Retrospective Evaluation of Implants Placed in Iliac Crest Autografts and Pristine Bone. J. Clin. Med..

[13] Kloss F.R., Kämmerer P.W., Kloss-Brandstätter A. (2022). Risk Factors for Complications Following Staged Alveolar Ridge Augmentation and Dental Implantation: A Retrospective Evaluation of 151 Cases with Allogeneic and 70 Cases with Autogenous Bone Blocks. J. Clin. Med..

[14] Dahl G. (1943). Om mojligheten for inplantation i kaken av metallskelett som bas eller retention for fasta eller avtagbara proteser. Odontol. Tidskr..

[15] Gore D., Frazer R.Q., Kovarik R.E., Yepes J.E. (2005). Vitallium. J. Long-Term Eff. Med. Implant..

[16] Linkow L., Wagner J., Chanavaz M. (1998). Tripodal mandibular subperiosteal implant: Basic sciences, operational procedures, and clinical data. J. Oral Implantol..

[17] Goldberg N.I., Gershkoff A. (1949). The implant lower denture. Dent. Dig..

[18] Linkow L.I., Dorfman J.D. (1991). Implantology in dentistry. A brief historical perspective. N. Y. State Dent. J..

[19] Bodine R.L., Kotch R.L. (1953). Experimental subperiosteal dental implants. US Armed. Forces Med. J..

[20] Small I.A., Misiek D. (1986). A sixteen-year evaluation of the mandibular staple bone plate. J. Oral Maxillofac. Surg..

[21] Greenfield E.J. (1991). Implantation of artificial crown and bridge abutments. 1913. Int. J. Oral Implantol..

[22] Sandhaus S. (1968). Technic and instrumentation of the implant C.B.S. (Cristalline Bone Screw). Inf. Odontostomatol..

[23] Brånemark P.I. (1983). Osseointegration and its experimental background. J. Prosthet. Dent..

[24] Abraham C.M. (2014). A Brief Historical Perspective on Dental Implants, Their Surface Coatings and Treatments. Open Dent. J..

[25] Liu H., Xuan Gan M., Zhai W., Song X. (2023). Design and additive manufacturing of root analogue dental implants: A comprehensive review. Mater. Des..

[26] Cipollina A., Ceddia M., Di Pietro N., Inchingolo F., Tumedei M., Romasco T., Piattelli A., Specchiulli A., Trentadue B. (2023). Finite Element Analysis (FEA) of a Premaxillary Device: A New Type of Subperiosteal Implant to Treat Severe Atrophy of the Maxilla. Biomimetics.

[27] Cerea M., Dolcini G.A. (2018). Custom-Made Direct Metal Laser Sintering Titanium Subperiosteal Implants: A Retrospective Clinical Study on 70 Patients. Biomed. Res. Int..

[28] Ayhan M., Cankaya A.B. (2023). Custom-made Subperiosteal Implants: A Finite Element Analysis on Monoblock and Dual Implant Systems in Atrophic Maxilla. Int. J. Med. Sci..

[29] Mangano C., Bianchi A., Mangano F.G., Dana J., Colombo M., Solop I., Admakin O. (2020). Custom-made 3D printed subperiosteal titanium implants for the prosthetic restoration of the atrophic posterior mandible of elderly patients: A case series. 3D Print. Med..

[30] Carnicero A., Peláez A., Restoy-Lozano A., Jacquott I., Perera R. (2021). Improvement of an additively manufactured subperiosteal implant structure design by finite elements based topological optimization. Sci. Rep..

[31] Vatteroni E., Covani U., Menchini Fabris G.B. (2023). The New Generation of Subperiosteal Implants for Patient-Specific Treatment of Atrophic Dental Arches: Literature Review and Two Case Reports. Int. J. Periodontics Restor. Dent..

[32] Gerken U., Esser F., Möhlhenrich S., Bartella A., Hölzle F., Fischer H., Raith S., Steiner T. (2020). Objective computerised assessment of residual ridge resorption in the human maxilla and maxillary sinus pneumatisation. Clin. Oral Investig..

[33] Mommaerts M.Y. (2017). Additively manufactured sub-periosteal jaw implants. Int. J. Oral Maxillofac. Surg..

[34] Demenko V., Linetskiy I., Linetska L., Yefremov O. (2019). Load-carrying capacity of short implants in edentulous posterior maxilla: A finite element study. Med. Eng. Phys..

[35] Kaman S., Atil F., Tekin U., Ozgul O., Önder M.E., Yilmaz S., Gungor H., Kocyigit I.D. (2017). Stress Analysis of Zygomatic Implants on the Augmented Maxillary Sinus: Is It Necessary to Graft?. Implant. Dent..

[36] Liu X., Pang F., Li Y., Jia H., Cui X., Yue Y., Yang X., Yang Q. (2019). Effects of Different Positions and Angles of Implants in Maxillary Edentulous Jaw on Surrounding Bone Stress under Dynamic Loading: A Three-Dimensional Finite Element Analysis. Comput. Math. Methods Med..

[37] Furumoto T., Oishi K., Abe S., Tsubouchi K., Yamaguchi M., Clare A.T. (2022). Evaluating the thermal characteristics of laser powder bed fusion. J. Mater. Process. Technol..

[38] Chastand V., Quaegebeur P., Maia W., Charkaluk E. (2018). Comparative study of fatigue properties of Ti-6Al-4V specimens built by electron beam melting (EBM) and selective laser melting (SLM). Mater. Charact..

[39] Benedetti M., Fontanari V., Bandini M., Zanini F., Carmignato S. (2018). Low- and high-cycle fatigue resistance of Ti-6Al-4V ELI additively manufactured via selective laser melting: Mean stress and defect sensitivity. Int. J. Fatigue.

[40] Mertova K., Dzugan J., Roudnicka M. (2018). Fatigue properties of SLM-produced Ti6Al4V with various post-processing processes. IOP Conf. Ser. Mater. Sci. Eng..

[41] Yánez A., Fiorucci M.P., Martel O., Cuadrado A. (2022). The Influence of Dimensions and Powder Recycling on the Roughness and Mechanical Properties of Ti-6Al-4V Parts Fabricated by Laser Powder Bed Fusion. Materials.

[42] (2016). Dentistry—Implants—Dynamic Loading Test for Endosseous Dental Implants.

[43] (2017). Dentistry—Implants—Dynamic Loading Test for Endosseous Dental Implants (ISO 14801:2016).

[44] Fellows I 2012 Deducer: A data analysis GUI for R Journal of Statistical Software 49.

[45] (2022). Ensayo de Tracción de Materiales Metálicos.

[46] Anon Standard Test Methods for Tension Testing of Metallic Materials. Anon. https://www.astm.org/e0008_e0008m-22.html.

[47] Aparicio C., Ouazzani W., Aparicio A., Fortes V., Muela R., Pascual A., Codesal M., Barluenga N., Manresa C., Franch M. (2010). Extrasinus zygomatic implants: Three year experience from a new surgical approach for patients with pronounced buccal concavities in the edentulous maxilla. Clin. Implant. Dent. Relat. Res..

[48] Falkowska A., Seweryn A., Skrodzki M. (2020). Strength Properties of a Porous Titanium Alloy Ti6Al4V with Diamond Structure Obtained by Laser Power Bed Fusion (LPBF). Materials.

[49] (2020). Materiales Metálicos. Ensayo de Tracció.

[50] Baumers M., Dickens P., Tuck C., Hague R. (2016). The cost of additive manufacturing: Machine productivity, economies of scale and technology-push. Technol. Forecast. Soc. Change.

[51] Santecchia E., Spigarelli S., Cabibbo M. (2020). Material Reuse in Laser Powder Bed Fusion: Side Effects of the Laser—Metal Powder Interaction. Metals.

[52] Anon Standard Test Method for Determination of Oxygen and Nitrogen in Titanium and Titanium Alloys by Inert Gas Fusion. Anon. https://www.astm.org/e1409-13r21.html.

[53] Łoginoff J., Majos A., Elgalal M. (2024). The Evolution of Custom Subperiosteal Implants for Treatment of Partial or Complete Edentulism in Patients with Severe Alveolar Ridge Atrophy. J. Clin. Med..

[54] Kakar A., Kakar K., Sripathi Rao B.H., Lindner A., Nagursky H., Jain G., Patney A. (2018). Lateral alveolar ridge augmentation procedure using subperiosteal tunneling technique: A pilot study. Maxillofac. Plast. Reconstr. Surg..

[55] Roy M., Corti A., Dominici S., Pompella A., Cerea M., Chelucci E., Dorocka-Bobkowska B., Daniele S. (2023). Biocompatibility of Subperiosteal Dental Implants: Effects of Differently Treated Titanium Surfaces on the Expression of ECM-Related Genes in Gingival Fibroblasts. J. Funct. Biomater..

[56] Hong J.-Y., Ko S.-Y., Lee W., Chang Y.-Y., Kim S.-H., Yun J.-H. (2020). Enhancement of Bone Ingrowth into a Porous Titanium Structure to Improve Osseointegration of Dental Implants: A Pilot Study in the Canine Model. Materials.

[57] O’Connell J., Murphy C., Ikeagwuani O., Adley C., Kearns G. (2009). The fate of titanium miniplates and screws used in maxillofacial surgery: A 10 year retrospective study. Int. J. Oral Maxillofac. Surg..

[58] Yanase R.T., Bodine R.L., Tom J.F., White S.N. (1994). The mandibular subperiosteal implant denture: A prospective survival study. J. Prosthet. Dent..

[59] Rams T.E., Balkin B.E., Roberts T.W., Molzan A.K. (2013). Microbiological Aspects of Human Mandibular Subperiosteal Dental Implants. J. Oral Implantol..

[60] Kochar S.P., Reche A., Paul P. (2022). The Etiology and Management of Dental Implant Failure: A Review. Cureus.

[61] Rinaldi M., De Neef B., Loomans N.A.J., Mommaerts M.Y. (2020). Guidelines for the Use of Resection Guides for Subperiosteal Maxillary Implants in Cases of Terminal Dentition—A Novel Approach. Ann. Maxillofac. Surg..

[62] Yánez Santana M.A., Fiorucci M.P., Cuadrado Hernández A.J., Martel Fuentes O., Monopoli D. (2020). Surface roughness effects on the fatigue behaviour of gyroid cellular structures obtained by additive manufacturing. Int. J. Fatigue.

[63] Bagehorn S., Wehr J., Maier H.J. (2017). Application of mechanical surface finishing processes for roughness reduction and fatigue improvement of additively manufactured Ti-6Al-4V parts. Int. J. Fatigue.

[64] Fatemi A., Molaei R., Sharifimehr S., Phan N., Shamsaei N. (2017). Multiaxial fatigue behavior of wrought and additive manufactured Ti-6Al-4V including surface finish effect. Int. J. Fatigue.

[65] Velasco-Castro M., Hernández-Nava E., Figueroa I.A., Todd I., Goodall R. (2019). The effect of oxygen pickup during selective laser melting on the microstructure and mechanical properties of Ti–6Al–4V lattices. Heliyon.

[66] Yan M., Dargusch M.S., Ebel T., Qian M. (2014). A transmission electron microscopy and three-dimensional atom probe study of the oxygen-induced fine microstructural features in as-sintered Ti–6Al–4V and their impacts on ductility. Acta Mater..

[67] Sidambe A.T. (2014). Biocompatibility of Advanced Manufactured Titanium Implants—A Review. Materials.

[68] Carrion P.E., Soltani-Tehrani A., Phan N., Shamsaei N. (2019). Powder Recycling Effects on the Tensile and Fatigue Behavior of Additively Manufactured Ti-6Al-4V Parts. JOM.

[69] Ayna M., Gülses A. (2022). Adapting a simple surgical manual tool to a 3D printed implantology protocol: The use of a universal screwdriver for fixation of custom-made laser sintered titanium subperiosteal implants. 3D Print. Med..

[70] Ishak M., Kadir M., Sulaiman E., Abu Kasim N. (2013). The application of the FE method by using a Computer Aid Engineering (CAE) tool as Ansys is an instrument widely spread in many engineering disciplines for an extensive variety of purposes. Int. J. Oral Maxillofac. Implant..

[71] Saini H., Ackland D., Gong L., Röhrle O. (2020). Occlusal load modelling significantly impacts the predicted tooth stress response during biting: A simulation study. Comput. Methods Biomech. Biomed. Eng..

